# **C**hallenges in Paediatric Xenotransplantation: Ethical Components Requiring Distinct Attention in Children and Obligations to Patients and Society

**DOI:** 10.1007/s11673-024-10377-5

**Published:** 2024-10-10

**Authors:** Anthony Merlocco, Daniel J. Hurst

**Affiliations:** 1 Department of Pediatrics, Division of Cardiology, University of Tennessee Health Science Center, Memphis, TN USA; 2 LeBonheur Children’s Hospital, 49 N. Dunlap St, 3rd Floor FOB, Memphis, TN 38103 USA; 3 Department of Family Medicine, Rowan-Virtua School of Osteopathic Medicine, Stratford, NJ USA

**Keywords:** Xenotransplantation, Paediatrics, Genetically modified organisms, Informed consent, Mental health, Public health

## Abstract

The transplantation of non-human organs into humans, or xenotransplantation (XTx), has recently garnered new attention and is being developed to help address the problem of organ scarcity in transplantation. Ethical issues surrounding XTx have been studied since initial interest arose decades ago and have experienced renewed discussion in the literature. However, the distinct and relevant differences when applied to children has largely been overlooked with few groups attending to the concerns that XTx in children raises. In this paper, we explore ethical challenges to be expected in paediatric XTx, in particular exploring organ sizing concerns, infectious risks, psychological burdens, and issues of moral hazard. We review these domains with the aim of highlighting the implications of pursuing paediatric XTx and the cross-disciplinary approach needed to solve these issues. Children require a unique analysis from a bioethical perspective to best prepare for the issues XTx presents.

## Introduction to Variation of Ethical Components in the Initiation of Adult and Paediatric Xenotransplantation

Efforts of cross-species transplantation, known as xenotransplantation (XTx), have been recorded throughout the eighteenth, nineteenth, and twentieth centuries, with examples spanning from blood to tissue and skin and even to chimpanzee kidneys transplanted into humans (Cooper et al. 2015). Swift rejection by the body’s immune system generally halted the use of animal organs, and ethical arguments against experimentation and futile care (given the perceived inevitable rejection) as well as the availability of alternative treatments such as kidney dialysis led to various calls for voluntary moratoriums on XTx (Institute of Medicine 1996; Tallacchini 2011). Renewed interest in XTx arose after the emergency use authorization for the first solid organ XTx of the twenty-first century in January of 2022 wherein a pig-to-human heart transplant was completed (Maschke et al., 2022). While this patient later died, it was not clear whether this was actually from organ rejection (Griffith et al. 2022). Development of bioethics perspectives in XTx is thus also gaining greater focus, yet the published literature to date has focused on adults rather than the paediatric sphere. XTx research in adults is currently underway but whether this should proceed in children may require rethinking ethical guidelines for first-in-man trials, though others have argued that because children participate in other experimental therapies we should consider paediatric XTx trials now (Hurst, et al. 2021a, b; Cleveland et al. 2019).

Children who are in need of heart transplantation experience significant morbidity, including when supported by mechanical support devices such as ventricular assist devices (VAD), with end-organ dysfunction, sepsis, and stroke affecting upwards of forty percent of patients (Hollander et al. 2021). Increasing organ availability and decreasing waitlist time is thus of critical importance for not only survival but decreasing morbidity. Others have argued that paediatric patients may be the most in need of a viable alternative, especially given waitlist mortality rates of up to 30 per cent (Goldstone et al. 2023).

Considerable barriers in the scientific achievability of XTx have previously made the ethical issues conceptual and seemingly remote. Significant advances have brought issues to the forefront, such as 1) harm evaluations arising from interspecies disease transmission and non-maleficence considerations for population health, 2) questions surrounding research ethics, consent, and long-term research obligations, and 3) policymaking for allocation, costs, and equitable access and distributive justice concerns. These ethical issues may be shared between adults and children but there will also be variation due to unique ethical context in the initiation of XTx and the solutions required. There has been very limited discussion of the ethics of paediatric XTx. Mainly there has been focus on how to approach the right to withdraw from clinical trials in this population and whether legislation should accompany XTx clinical trials to ensure follow-up (Hurst et al. 2020; Merlocco 2023). Encouragingly, a recent consensus expert opinion statement from The Journal of Thoracic and Cardiovascular Surgery, while focused on the clinical aspects of paediatric XTx, recognized that “all aspects of research, clinical-scale production of organs, and clinical translation require an utmost ethical scrutiny” (Konstantinov et al. 2022, 964). Beyond the unique consent considerations, psychosocial implications and risk of experimentation or non-benefit have been raised but require further exploration (Padilla et al. 2023). Ethical issues facing paediatric xenograft candidates or recipients may be the same as those facing adults but will have distinct considerations to be addressed within the paediatric domain and understanding them requires a renewed focus. While many of the issues we will discuss apply to paediatric XTx generally, we will present these considerations using cardiac XTx as a paradigm. We provide an overview of several reasons that children require a distinct ethical analysis, primarily using a risk–benefit framework, and when appropriate, apply a principlist view of how this framework might differ for children as compared to adults.

## Ethical Domains Requiring Consideration in Children: Justice and Risk in Sizing, Xenozoonosis, and Psychological Considerations

In Sect. "Control of Organ Growth, Increased Availability of Variable Organ Sizes, and Issues for Growing Patients", benefits from XTx organ size control strategies are examined. However, as organ growth engineering is a central aim of XTx development, this claim is brought into question, and we point out the potential that children may suffer harm from limited organ longevity due to arrested organ growth. In Sect. 2.2, two ethical concerns arising from exposure to novel animal pathogens (xenozoonosis) with differing potential harms to recipients and to society are examined. In Sect. "Risk of Xenozoonosis (Non-Human Animal Pathogens) in Paediatric Patients", the connections between psychological factors influencing identity, behaviour, graft risk, and re-transplantation are examined.

### Control of Organ Growth, Increased Availability of Variable Organ Sizes, and Issues for Growing Patients

Organ *size* at time of transplantation has always been an important consideration in allotransplantation. Organ *growth* over time after allotransplantation of a *human* organ is rarely considered because after size-matching, donation between humans involves organs that do not need to grow or that will grow commensurate with patient growth. Organ transplantation has always required consideration of organ size similarity between donor and recipient. Size matching is integral to optimizing outcomes (Eberlein and Reed 2016), and oversized organs demonstrate dysfunction with fewer donors being feasible (Tuba and Ali 2020). In paediatric recipients this results in a narrower range of acceptability. Paediatric XTx is proposed to allow optimal organ sizing for any age (Cleveland et al. 2019; Hinrichs et al. 2021; Reichart et al. 2021). However, a significant and overlooked issue with potential to do harm centres on organ growth uncertainty. Actually, a significant concern in adult XTx is unregulated post-XTx organ growth resulting in hypertrophy, dysfunction, and failure, unlike after allotransplantation of a human organ. The solution has been *to arrest* growth, which reduces not only the donor organ size but growth velocity and animal size at maturity (Wall et al. 2020; Längin et al. 2018; Hinrichs et al. 2021). While growth-arrested organs may be a goal in adults, they may limit benefit in children, particularly in younger children due to a rapid growth velocity. Of particular concern, if a “matured” organ cannot continue to grow and is transplanted into a growing (paediatric) recipient, the child will quickly outgrow the organ. If XTx is utilized as a bridge-to-allotransplantation of course this concern is mitigated as the organ longevity may not need to extend to years, however this balance of longevity and growth requires attention given that various goals of XTx have been proposed.

Ethical analysis of how to balance the risks inherent in paediatric XTx against the potential benefit is underexplored. Specifically, it is not clear if the benefit is more than short-term solution without solving the organ growth question. Certainly, saving a child’s life and providing a functioning organ is a significant benefit, but this is not necessarily true if the organ can only achieve this goal for a short time, especially when our knowledge of the XTx risks remains limited. The prudential value of growth-arrested XTx in children is thus difficult to assess because we have not addressed the scope of this issue. Whether paediatric patients will benefit from availability of smaller organs remains uncertain since cessation of growth would limit longevity of the organ in a growing child. Yet, while the benefit of survival will fade as the organ size quickly becomes insufficient, this does not mean XTx is of no value—indeed, extending survival until another option may be feasible (i.e. using XTx as a strategic “bridge” to a human heart transplant or until a child is large enough for more mechanical support options) may change the ethical evaluation. While the strategic goals of XTx in children are clear: provide XTx that can grow with the child, or provide XTx as a bridge with an appropriate post-XTx plan, the feasibility is much less clear and thus shifts the ethical balance when compared to adults for whom neither issue above exists. This issue has largely escaped not only responses but even recognition with research often conflating potential benefits or harms for growing patients. Conflictingly, one group points out that, “this strategy may also be important to provide xenohearts for pediatric patients,” (Hinrichs et al. 2021, 8) however they also argue that a “clear advantage of [growth-limited] pigs is that the donors can be used at an older age when they have already passed the steepest phase of their growth curve” (8).

There is limited data upon which to make risk and benefit assessments. Baboons have been studied, with similar weight to human children of 15–30 kg, but as these were adult baboons, *growth* questions cannot be answered (Goerlich et al. 2021). Consider the longevity and growth required for a heart transplanted into a three kilogram neonate, whose median survival after transplantation is 20.7 years (Dipchand 2018). Initially, very significant growth is required to remain commensurate with the patient’s weight, which doubles in the first six months and triples by one year (Waseem et al. 2021). It is not yet clear whether “growth-restricted” will mean only limiting hypertrophy or limiting full somatic growth following changes in body dimensions.

The ethical issues centring on how patients access waitlists or with how providers counsel, care for, and plan post-transplant care have been discussed extensively in the allotransplantation realm, however growth-arrested XTx organs may require a novel analysis in the context of an anticipated need for re-listing, particularly for its effects on non-maleficence and justice considerations. More limited longevity would require potential planning for re-listing and waitlist prioritization compared to primary allotransplantation. Post-xenotransplant care will also involve known periods where decompensation can be predicted and, while there may be advantages to knowing when and how a patient will become ill, since this is induced by the novel XTx strategy itself, it must be weighed against waiting for allotransplantation. As this balance is unknown, the principle of non-maleficence may be prioritized when considering a growth-restricted organ. An additional issue is whether patients stabilized by XTx could lose allotransplantation waitlist prioritization status due to improved health. Justice arguments can be made in both directions in that a patient should not take a waitlist position if they have a transplanted organ. Yet on the other hand they are taking on risks shared between XTx and allotransplantation while failing to receive the total benefit of many years of a functioning graft associated with allotransplantation. Especially in the context of a clinical trial with an experimental therapy, it would seem unethical to take a patient off an allotransplantation waitlist while safety and efficacy remain uncertain.

A potential response may be simply to make two groups of donor pigs, one with growth hormone receptor knocked out and the other intact and utilize the latter in children so that the organ grows. However, this grossly oversimplifies and misconstrues the issue. Post-transplantation growth dysregulation is not simply a problem of organ size but rather involves multi-factorial issues with tissue distribution, organ mass, and function. Specifically in the heart, when xenograft growth is dysregulated, the hypertrophied muscle cannot relax and results not only in poor heart function but upstream and downstream organ dysfunction of the lungs and liver and ultimately reduced graft survival (Goerlich et al. 2021).

It is important to note that because paediatric XTx trials have not begun, strategies for organ-growth control have not yet been determined. There has been work with porcine XTx models that does not utilize growth knockout but the growth in size and weight can be extensive and unregulated but other strategies such as rapamycin may balance these concerns (Litovsky et al. 2022; Iwase et al. 2021).

Growth-control of XTx organs will certainly influence the value for paediatric patients but work remains to be done on improving the strategies and the main advantage for children may still be greater availability of a narrow range of organ sizes without waitlist delay and as a possible “bridge.” We point out, though, that if this organ cannot grow, these children will be required to take on many of the risks of XTx while the benefit of a functioning organ may be limited, which has not yet been addressed in the literature. Our concern is that normal organ growth and dysregulated hypertrophy likely follow different mechanisms and the organ growth issue currently remains unresolved and should elicit more discussion if paediatric trials are considered. Growth rates may vary and so we do not discuss this as a reason not to pursue paediatric XTx but rather to highlight the knowledge gaps in how somatic growth may be affected and what strategies may need to be employed if uncontrolled hypertrophy is also a concern.

Relevant issues surrounding whether XTx organs can serve all paediatric sizes and the differences in growth velocities will influence how to approach XTx candidacy, counselling, wait-listing, and management. Not all children may benefit equally from research efforts. The timing of diagnosis and “made to order” organs will differ depending on recipient age, which may result in cost and availability inconsistencies if all sizes are meant to be available at all times. If we accept this as a relevant moral concern, then a duty arises to solve the issue of equitable provision of organs of variable sizes. If we do not consider this a duty, then paediatric patients may have few XTx options or increased wait-times for organs compared to adults. If technological advances solve the growth problem, this would allow children swiftly to benefit due to limited treatment options currently available to them for failing organs.

### Risk of Xenozoonosis (Non-Human Animal Pathogens) in Paediatric Patients

Transplant populations are at particular risk of xenozoonosis (cross-species disease) because of exposure to unique pathogens and required post-transplant immunosuppression. Additionally, because the paediatric immune system is developing, risk may be compounded. Two ethical concerns arise with potential introduction of these pathogens into a transplant recipient: 1) will recipients be at risk of greater harm than conferred by allotransplantation, and 2) can the benefit of XTx to an individual justify the potential infectious risks to the non-recipient human population by transmission from recipients to the non-recipients. A previously naïve population may be at particular risk for novel disease and even an epidemic. One reason that concerns with xenozoonosis in XTx are significant is that the animal pathogen has been *directly inoculated* into the human host, whereas with concerns for non-XTx xenozoonosis, the *environmental* exposure is likely not comparable. Current animal research protocols to reduce the risk of transmission are based on what is already known about working in close proximity to animals. Fewer examples exist of direct insertion of animal tissue upon which to base precedent and policy. Precisely because it is tied to recipient interests, but any anticipated risks are poorly quantified, it deserves special consideration of benefits and harms, and we argue for a principlist approach.

Additional concerns have also been presented that efforts to raise disease-free source animals are in tension with animal welfare and best practices of animal care (Johnson 2022). Animal well-being and suffering already creates ethical conflict among veterinarians (Moses et al. 2018) and is less considered in the research realm, however we will limit ourselves to ethical arguments balancing patient benefit and public risk in the context of potentially unique hazards conferred by a paediatric immune system. We must consider the possible harm associated with a potentially increased risk of mutation and widespread transmission when determining how the most patients can benefit from XTx safely.

#### Potential Xenozoonosis and Harm to the Paediatric Recipient

A pig organ could potentially contain a pathogen either that is similar to common human pathogens or that is a unique pathogen without precedent in humans and, importantly for either, there are few or no detection methods and the risks to humans are unknown (Fishman 2018). Thus, surveillance and prevention must focus on rarely encountered pathogens that may not currently even be detectable. Efforts to grow animals that are germfree or have completely defined flora are difficult, labour-intensive, and costly and may not even account for endogenous or previously uncharacterized viruses (Oklahoma Medical Research Foundation 2022).

The ability to reliably create pathogen-free animals remains in question and whether this is even possible remains central to harm evaluations and how to assess beneficence and non-maleficence to make appropriate exposure choices. Significant efforts in pig husbandry and bio-secure housing of genetically modified pigs were required for the first pig-to-human heart XTx in order to control multiple porcine viruses not seen in humans such as porcine cytomegalovirus (pCMV). Importantly, while initial testing of the animal and organ demonstrated no pCMV, it was detected 20 days after transplantation and was determined to have been latent in the pig heart (Griffith et al. 2022). Despite significant and innovative infection control efforts such as advanced animal husbandry practices, PCR surveillance, and anti-viral prophylaxis, they note, “detection of pCMV was unexpected” (Griffith et al. 2022).

Human exposure to animal tissue has a long history and while some viruses are able to infect human cells, such as porcine endogenous retrovirus (PERV), it is not via normal environmental exposure (Patience et al. 1997). While animal tissue exposure may not be novel, infection remains incompletely understood. In patients exposed to living pig tissue by graft, perfusion, or porcine islet cell transplantation, PERV infection was not detected though persistent porcine cells in the blood were observed for several years (Paradis et al. 1999). This may be encouraging, however the combination of exposure to animal pathogens *and* immunosuppression required for XTx is difficult to factor, especially since not all pathogens may be equally infectious. As well, the immunosuppression for XTx is unique from standard allotransplantation regimens (Sykes and Sachs 2022). This may also mitigate our concern for the general population exposed to xenozoonosis from XTx recipients since most individuals will be immunocompetent, however that should not undermine the justice concerns for immunocompromised persons who have no input in their exposure to xenozoonosis from XTx patients.

Paediatric immune systems have meaningful differences from adult immune systems that may confer greater xenozoonosis risk. The immune system evolves “over the lifetime of an individual,” and while, “the older individual will have encountered and established a memory bank to many pathogens over its lifetime,” paediatric patients have far fewer established exposures and defences (Simon et al. 2015). The paediatric innate and adaptive immune systems confer less protection from viruses, bacteria, fungi, and parasites (Simon et al. 2015). Children thus may 1) be more susceptible to disease and 2) be more likely to facilitate post-infection mutation resulting in the potential for novel human diseases and potentially pandemics.

While there is likely an increased risk in children, as with many of these considerations, it is difficult to quantify, and thus to weigh appropriately. This argument aims to convey that while the risk may be low, it is difficult to quantitate given the novelty and lack of precedent and the youngest children may require the most unique consideration. Interestingly there are also immunologic reasons that support children, particularly neonates, as XTx candidates due to and immunologic advantage against rejection (Carlo and Cleveland 2023). Weighing the immunologic concerns for evolving immune function against an immunologic advantage or other considerations to do the most good and least harm is difficult given uncertainty on the strength of these effects and the risk.

#### Potential Xenozoonosis and Harm to Society

The potential benefit of XTx to an *individual* is clear—prolonged life and decreased symptoms. Individual patient interests and societal welfare may potentially be at odds however if there is an increased risk of pathogen introduction to the population causing novel disease in non-recipients and, in the extreme, a new pandemic. There are good arguments that a society, in general, benefits from more organs and transplantation (Beard et al. 2013), but we will not be addressing that here. Rather, we argue that uncertainty of population-level xenozoonosis risk makes this balance difficult and ultimately may provide an argument for mitigating this risk by favouring adult XTx development while collecting more biosafety data.

If the risk of population-level xenozoonosis from paediatric XTx is minimal or no greater than current risks posed by animals living in proximity to humans, restriction of XTx would needlessly deny a great benefit. Yet, if the risk is significant then it may not be fair to expose others to such an infection, especially without population consent. With regard to adult XTx this has been raised elsewhere as individual consent to transplant is in tension with public health threats (Johnson 2022). Clearly this can be expanded to an argument against paediatric XTx, especially if consent in this population is limited. Here we will explore reasons why children may increase our concern and may require more data for mitigation strategies.

The basis for concern lay in whether paediatric patients, by virtue of their immune system characteristics, are more likely to be an agent for pathogen mutation that would confer greater risk to non-recipient persons than they currently experience. The strength of this concern is unknown because firstly, it is difficult to discern the probability of this transmission itself^1^ and secondly, the disease severity is unpredictable since any pathogens currently studied in animals would likely manifest differently in humans and mutations would change this as well. The evolving immune systems of children compared to those of healthy adults may place them at increased risk. In patients who are immunosuppressed, the risk of pathogen mutation is higher, resulting in novel disease in the human population with, recent observations in the COVID-19 pandemic that “highly mutated variants … preferentially occur in the milieu of partial immune control” (Corey et al. 2021, 562). Younger children, even those who are not immunosuppressed, demonstrate differences in immunity and require time to develop immunologic memory and are at risk of viral, bacterial, and parasitic infections (Simon et al. 2015). Since children, specifically young children, have a developing immune system, any potential, even conceptual, for an increased risk of mutated variants should elicit caution.

Arguments centring on the particular concerns of xenotransplantation apply both to adults and children. However, given the conceptual increased risk of mutation in children issues with pathogen detection gain greater weight—it may take more time to discover pathogens with more mutations and more difficult to develop assays. While some currently known pathogens are easily detectable with readily available assays, others may be more difficult to detect, or completely undetectable if they are yet unknown. Screening of source animals and subsequently their organs at the time of transplantation is only effective if we have tests for every pathogen and thus is inherently limited. Ascertaining that all pathogens have been ruled out may be an impossibility. In the case of the first pig-to-human cardiac XTx, efforts to minimize risk have resulted in source organs thought unlikely to cause disease but that nonetheless subsequently demonstrated latent infection (Griffith et al. 2022). The xenograft recipient died and while the mechanism remains under investigation, it is thought not to be related to rejection, raising concerns that pathogens may have played a role (Griffith et al. 2022).

Some non-recipients (e.g., friends, family members) may implicitly, or even explicitly, consent to exposure of a pathogen (assuming they are aware of the risk) through their association with the xenograft recipient. However, most members of society have no input beyond public policy feedback and are likely even unaware of the risk. If paediatric XTx were to confer a greater risk to society of pathogen transmission, then justice concerns would argue for further caution than already considered for adult XTx. The World Health Organization (WHO) has continually advocated for public input on the XTx regulatory system: “The regulatory system should be transparent, must include scientific and ethical assessment and should involve the public” (First WHO Global Consultation on Regulatory Requirements for Xenotransplantation Clinical Trials Changsha, China, 19–21 November 2008, 2009). How, and to what extent, the public should be involved is subject to interpretation. It could be argued that public input is not necessary, but this is balanced against the likelihood of harm to the public, which is conceptual. One ethical justification for ignoring public input may be based in arguing that humans are exposed to animals all the time and thus pathogens can always bypass the species barrier. Notably, there are currently no *official* prohibitions on having immunocompromised patients work with animals for their own protection or for that of society. Some precedent exists in guidelines for safe pet ownership and recommendations against animal laboratory employment (Kansas State University Compliance Office 2022; Steele 2008). However, the argument that XTx should not warrant any special attention fails to account for how little is known about the relative risk.

The public generally is not able to weigh in or contribute to measures to decrease risk of cross-species disease transmission and so policy decisions around XTx may represent a unique point of input. Whether the public should have input in “consenting” to a new world is a difficult question. Other scientific advances often evaluate public opinion, but effective input is more difficult. For example, for gene editing, “there is limited understanding among the groups calling for public engagement … about 1) the goals of this engagement, 2) the modes of engagement … and 3) how to connect” (Scheufele et al. 2021, 1). For XTx, public engagement should be a priority. Effective public input may be limited without effective engagement, education, and awareness in addition to difficulties coordinating international regulation and thus, while we do not argue for the prohibition of XTx, mitigation is important since the public may have little input into how XTx policy influences its risk and health, particularly for paediatric XTx.

The necessity for “harmonizing individual and collective rights” and understanding “links between individual consent, family consent, and acceptance by society” places XTx in a unique position for evaluation of public health protection approaches (Tallacchini 2011, 171). Where does the consent-requirement web end? As the xenograft recipient, as well as their immediate contacts, will continually be interacting with new contacts, this creates a situation of infinite regress where an ever-increasing number of persons must consent to the potential risk. The risks placed on family members, restrictions on individual freedom, and the creation of new societal risks requires a system “capable of organically connecting individuals and society at large” with “solidarity … and social cohesion … of all parties involved” (Tallacchini 2011, 178). Full societal explicit consent may be complex and not feasible, but there is a role for the recipient’s family and friends to provide informed consent “not as a potential veto on patient consent, but instead as an element of a broad social negotiation on the acceptance of a high risk procedure” (Tallacchini 2011, 178). An in-depth discussion of the spectrum of consent and assent is beyond our scope, but explicit consent may not need be the goal. Implicit consent, or even assent, may be appropriate wherein information is provided and questions answered but without a formal signature process. For their part, “well-informed citizens” surveyed about XTx are “willing and interested to get involved … and arrive at a well-informed and carefully reasoned evaluations” (Kögel and Marckmann 2020, 3), though this is without detailed discussion of paediatric xenozoonosis considerations. Importantly, while supportive, they held the, “conviction that technologies such as XT must have the approval of society, not merely the patient” (Tallacchini 2011, 178). Public input will be an important consideration as xenozoonosis risks become better studied and quantified among specific populations.

### Psychological Considerations—Mental Health Concerns (Identity and Bullying), Moral Hazard and Behaviour, and Re-transplantation

Due to developmental and psychosocial immaturity, children may be more susceptible to harms related to the perception of “unnaturalness” of animal organs. Children may be less well equipped to manage the emotions and identity shifts that come with the *idea* of becoming “part human-part animal,” even if we reject that idea that XTx actually causes an ontological change to somehow “part-animal.” As well, if news of a child’s XTx extends to peers, they may experience bullying and teasing, especially if XTx is very novel. This concern was voiced by parents of children with congenital heart disease in one single centre study (Hurst et al. 2021a, b). Additionally, there is a risk that with unlimited organs, patients will become poor stewards of their organs, a form of moral hazard, which could drive more transplantation overall due to increased rates of rejection. Children are still developing in forethought and accountability and may be likely to act recklessly. While none of these provide a strong enough reason to prohibit paediatric XTx, multi-disciplinary approaches to address concerns proactively are needed.

#### Identity, Mental Health, and Bullying

Discussion of the many senses of “identity” is beyond our purpose here but the idea of self-conception as “personal identity” will be employed using a framework argued by Mark Schroeder that personal identity is narrative in nature and considering this in the “strict sense of identity—the question of which person you are, and how that person is extended in space, time, and quality” (Schroeder 2022, 209). The experiences of a pre-adolescent or adolescent particularly can shift the trajectory of personal identity development. Experiences may significantly transform identity if the effects are strong enough and we would argue that in particular for children, both the experience of *undergoing* an animal organ implantation as well as *living with* that non-human organ inside presents a very strong effect.

There is little precedent upon which to base how a person will feel with a *complete non-human animal organ* implanted. Chemically treated non-human animal tissue has an extensive history of implantation for heart valves for both adults and children but this generally is referred to simply as a “heart valve” with little focus on its origin. XTx of a heart may elicit a significantly different perception however. XTx of a heart does not need to *actually* change any meaningful ontological features of the recipient. Merely the *perception* of a potential ontological change could cause identify shifts and consequent distress. While theoretical, there are grounds for the strong and persistent conceptualization that “the heart is where personality resides and is the locus and seat of emotions, thought, and will” (Rollin 2020, 8). In general then, replacement of an organ may engender a sense of “organismic discontinuity” (to borrow from artificial intelligence literature), referring to “an organism view of personal identity … [wherein] we persist … so as long as organismic continuity is maintained” (Liao 2020, 491). We need not even adopt this formulation to raise concerns if we use the narrative identity approach because even if one does not accept that XTx results in organismic discontinuity (which indeed it may not) a child need only *feel* as though this discontinuity has somehow occurred and they are “part-animal.”

Bernard Rollin argues that the strength of the link between the psychological and physical conceptions of self will drive “opposition to xenotransplantation [that] stems from the appalling degree of social illiteracy regarding transplant science” (Rollin 2020, 8). This raises a valid public education point and Rollin expresses concern for the.

“belief that if one acquires a heart from a pig, one will have the emotions and feelings of a pig, and thus be rendered considerably less than human” (Rollin 2020, 8).

It remains unclear if Rollin’s concern would be widespread or substantial. While literature in this realm is lacking, for their part, recipients of *non-living* animal tissue such as pig-heart valves have seemed to cope well. Albeit without specific opinions expressed about self-concept, both paediatric and adult patients experience “renewed hope” and “regained independence” with these procedures (Andresen et al. 2014). Of course, it is unclear if patients still consider these valves “pig-like” after chemical treatment or how informed children may be of the tissue origin. When a child receives a currently available pig non-living *tissue* heart valve, it is not clear whether the child themselves or their peers are aware that the heart valve is of non-human origin.

In contrast to inert, chemically treated pig-heart valves, paediatric awareness of the animal origin in XTx will be nearly unavoidable given requirements for surveillance, research, and informed consent and assent. Children are more immature and vulnerable and may be particularly at risk of distress given an increased attention in the lay press on the “animal” nature of these xenografts. With XTx however, two linked concerns arise: 1) increased attention *in general* in the press regarding how “different” these animal transplants are, and 2) whether an individual child will be aware of their own XTx origin. Rollin points out exactly this concern when addressing privacy, stating,

… it is extremely unlikely that the identity of those receiving xenotransplants can be kept confidential, so it is quite possible that such people will be seen as significantly different, or even as “freaks,” particularly if the recipient is a child and will likely be subject to taunting with locutions like “pig heart” regularly thrown at them, which in turn can cause significant psychological damage … (Rollin 2020, 3)

While this may be a pessimistic view of the schoolyard, it warrants attention and, even if peers are unaware, the child themselves will likely be aware and should be counselled because of public perceptions of XTx that may generate emotional distress at being “part-animal.” One argument may advocate for keeping children uninformed to control and protect their experience. While this would be short-lived anyway due to the required XTx follow-up through adolescence and adulthood, paediatric bioethicists generally accept that children should be told the truth when developmentally appropriate, even independent of any evaluation of decision-making participation (Hudson et al. 2019). XTx should be no different, especially given the increased demands and surveillance.

Cardiac surgery and transplantation is already associated with psychosocial distress in children (De Pasquale et al. 2020; Di Matteo et al. 2018; King et al. 2009; Society of Pediatric Psychology 2022; Younes et al. 2019), with over a quarter experiencing “clinically significant emotional/behavioural adjustment difficulties…heightened anxiety, mood dysregulation and…reduced self-esteem, isolation and academic difficulties” (Society of Pediatric Psychology 2022). It is on this background that we should proceed with caution in those more vulnerable when the psychological effects of receiving a pig-heart are unknown and may foreseeably induce distress. Public acceptance and a sense of community will be critical components of mitigating these concerns with efforts “aimed both at strengthening … feelings of belonging to imagined XTx communities and at blurring the boundaries between swine and humans, depicting the pig as ‘one of us’”(Tallacchini 2011, 182).

While these psychological effects hardly preclude paediatric XTx given the potential benefit and that the alternative could be death, they represent credible challenges. Current studies advocate generally for psychiatric consultation surrounding cardiac surgery (Younes et al. 2019) and XTx will present a new situation requiring novel and increased planning with multidisciplinary approaches. We argue that the shifts in personal identity will require more than the current psychological pre- and post-allotransplantation counselling. A multi-disciplinary approach is integral, but likely will need to incorporate a broader spectrum of perspectives, training, and fluency in personal identity ethics. This may be accomplished by further consultation with not only the hospital ethics committee but specific education and counselling development by ethicists and philosophers working with clinicians, psychologists, child life specialists, and families. This may take the form of interviews and discussion to ascertain how the child currently constructs their identity, how influential positive and negative experiences will be in shifting this, and how to anticipate managing peer reactions and interactions.

#### Moral Hazard and Re-transplantation

XTx may increase the likelihood of moral hazard effects leading to poor organ stewardship and the need for re-transplantation. These considerations must be addressed particularly in children given their developing maturity and reasoning aptitudes. Organ stewardship is important for recipients of any age, but children are more likely to lack the understanding and experience to engage the requisite sense of responsibility, maintain accountability, and restraint from reckless behaviour. One may argue that the crucial advantage of XTx is that organs are plentiful, however, many other resource constraints, healthcare costs, and organ rejection considerations still provide good reason to remain a good steward. In particular, if xenografts are rejected, even if another is available for re-transplantation, the recipient’s suitability may change due to prior XTx exposure and post-transplantation management. As well, supporting and fostering responsibility, particularly in patients with chronic disease has both prudential and moral value and we should avoid devaluation of this aim that may arise from conceptual resource abundance. It is important to point out here that non-adherence to post-transplant care is complex with many factors, including social determinants of health, contributing. However, in addition to these well-documented and studied factors, XTx and the public perception of XTx may increase the likelihood and effect or moral hazard and it may do this disproportionately in children.

“Moral hazard” comes from the insurance industry, referencing when “policyholders take less care or do not minimize … risk of loss due to the fact that they have insurance to cover losses” (Prince and Schwarcz 2020, 1291). XTx creates a genuine moral hazard via the perception of readily available organs as a form of “insurance,” disincentivizing stewardship to minimize risk of rejection. As stated, there may be other resource considerations beyond merely the *number* of organs XTx potentially could provide, thus the argument centres on the *perception* of unlimited organs resulting in irresponsible behaviour or so-called moral hazard. Consequently, this may actually *increase* XTx demands for *re-transplantation* in children after xenograft failure, and so a central additional reason to address moral hazard is to prevent re-listing, whether in the XTx or allotransplantation pool.

Transition from the transplant waitlist to receiving a transplant is a joyous event but comes with significant responsibility. How a patient will respond to this responsibility is never known with certainty and listing for transplantation includes assessment of this factor. Adherence to medication requirements, follow-up plans, and healthy behaviours are integral to transplantation success. However, a concerning number of children who receive transplants become non-adherent, generally around the age of adolescence (Oliva et al. 2013; Shellmer et al., 2011) and this presents significant moral questions when assessing whether they can be re-listed for transplantation if their graft fails. Re-listing itself, of course, is not uncommon, though graft failure is significantly higher than for primary transplantation (Magee et al. 2007).

One may argue that even if organs were plentiful, the experience of going through a hospitalization for XTx as well as the post-operative care would be burdensome enough to counter the influence of moral hazard leading to poor behaviour. It is true that these children would already have experienced a heart transplant, and this will inform their behaviour but depending on the patient’s age at initial transplantation, current age, maturity, and forethought, it is difficult to predict if this would minimally mitigate moral hazard concerns or completely resolve them. While this may exist on a spectrum, it remains salient as a potential consideration in this population.

Generally, with organ re-transplantation we require a higher threshold for listing because, whether from behavioural or biomedical factors, the recipient’s body has demonstrated at least one graft failed, indicating some increased probably of future rejection. One argument when faced with this data is that XTx may be a *better* approach for those facing re-transplantation than placing them back in the allotransplant pool because it does not require use of a scarce human organ (Banks et al. 2020). This is compelling when finding first candidates to initiate XTx, however as XTx becomes more available and standard, one would hope for medical equipoise such that the XTx and allotransplant pools combine. This complicates the moral hazard risk though because on one side it is more likely since this would strengthen the perception that organs can be casually retrieved. On the other side, even if moral hazard becomes more likely, it appears to be less compelling if retrieving an organ-off-the-shelf becomes an eventual reality.

The harm of this moral hazard could result in, “the costs of risky behaviour … spread among a large number of people” (Brown et al. 2019, 123). Freedom to behave responsibly can be complex as, “factors outside the agent’s control may influence behaviour in such a way as to undermine her freedom” (Brown 2013, 695), and this may be particularly pertinent in children and young people. The interaction between freedoms and behaviours raises a difficult question about how “deserving” children (especially of different developmental levels) are of re-transplantation. The answer is beyond our scope, rather, we argue only that if perceptions of XTx make some risk-taking behaviours more likely, then addressing moral hazard where it is more likely to occur will better facilitate XTx initiation. A counterargument is that non-adherent behaviours, whether lifestyle-choice related or factors beyond one’s control such as the inability to present for appointments, is inevitable and at least XTx is a solution that buttresses survival to adulthood when one may become more responsible or in a better position to ensure follow-up. This is a strong consideration; however it hardly alleviates all concerns surrounding moral hazard, particularly the moral value of taking responsibility for self-care and graft survival and the resource constraints across animal organ production, procurement, and surgery. It is not equivalent to other lower-cost moral hazard sequelae such as needing a second set of stiches for a re-opened wound or needing a new cast after re-breaking a bone. As well, patients who do poorly after XTx may request or require placement on the allotransplantation list. Earlier WE made the argument that XTx prior to allotransplantation may be an advantage as a “bridge” however this only applied as a *planned* strategy. If a failed XTx results from irresponsible behaviour and requires re-transplantation, there are relevant moral differences in allocating an allotransplant to that patient subsequently. Importantly, although organs may be available through XTx, clinically, a patient hoping for re-transplantation may become unable to due to frailty of several rejections and multiple chest operations, however, their *perception* that another organ is readily available may influence their attitudes and behaviour and the issue of re-transplantation and transparency will be an important component of informed consent prior to XTx.

Addressing moral hazard in paediatric XTx will require recognition, planning, and counselling. In the context of the known risk for poor adherence post-transplantation in adolescents, it will be important to discourage risky behaviour or even just a lax attitude to medication adherence and follow-up. Attempts to avoid moral hazard should also recognize whether an agent has sufficient control over their health, which is limited but evolving through childhood. Rebecca Brown argues that:

… ensuring the consequences of an action fall only on the individual performing it [will help] develop the capacity for responsibility in children (if they are told they will be held responsible they may learn to take on the role of a responsible agent) … (Brown 2013, 696)

Given that there is no experience currently with paediatric xenotransplantation much of our ethical considerations necessarily remain speculative, however we have drawn on data from adolescent post-allotransplantation non-adherence and extrapolated to a reality wherein organs are not actually limited to bring the issue of moral hazard into focus. In addressing moral hazard in XTx there may be avenues to develop responsibility in children and we should proactively create strategies to combat moral hazard if developing paediatric XTx is to be successful.

## Conclusion

Analysis of the ethical components of XTx has progressed alongside the XTx breakthroughs, with discussion of safety, consent, and surveillance issues requiring particular consideration. Paediatric XTx has garnered little attention however and distinct components of the ethical issues of XTx that relate to children have scarcely been recognized. The sequelae of these ethical issues have important cross-disciplinary relevance for whether to develop paediatric XTx and if so, how best to do so. The bioethical analysis of XTx seems a natural and necessary focus with XTx more prominently in public awareness but paediatric XTx and its ethics require distinct research and demand special attention. It is important to highlight that these issues are important *considerations* but it does not follow that XTx is thus impermissible or that simply addressing consent issues will provide full ethical justification for paediatric XTx, as others have importantly pointed out after the first adult porcine cardiac XTx (Gyngell et al. 2023). Additionally, the specific genetic model that may best serve paediatric XTx has not yet been determined and so we hope that highlighting the considerations in this manuscript will help guide ethical and scientific progress to account for unique concerns in children.

Xenotransplantation raises important challenges to science and society propelling distinct approaches to issues in paediatric ethics. We have reviewed how the paediatric immune system differs from the adult and how this could impact the risks of infection to the patient and to society. We have also highlighted concerns regarding the probable psychological challenges posed by children and adolescents with an incompletely developed sense of responsibility, maturity, and reasoning ability. XTx prompts questions of identity and also can be perceived as an “easy” resolution to organ dysfunction because it does not suffer the main challenge in transplantation, which is organ availability. These elements may pose challenges to any recipient, and certainly there are many adults lacking in maturity. We have responded though that children may be more likely to lack the coping skills and experience required and would benefit from development of multidisciplinary strategies to pre-emptively recognize and resolve these issues.

Paediatric issues of consent and obligations for follow-up and research involvement have long been addressed in the ethics literature but rarely have required the extensive responsibilities, surveillance, and uncertainty entailed by XTx. Uncertainty looms large regarding safety and durability, trial design, and future treatment alternatives. While it may be easy to recognize these concerns, finding resolutions will be challenging. In children though, these issues require greater recognition but there are compelling reasons to advocate for paediatric XTx. This paper advocates as such and provides justification for further study into the empirical components that will better inform how to approach these ethical issues, and which supports should be prioritized.

## Data Availability

Not applicable.

## Footnotes

1 Another consideration for probability of transmission is also how different countries regulatory approaches to surveillance and isolation may differ since this could vary widely. Of course, once in the human population, disease does not respect borders so transmission itself may be difficult to model.
